# Nutritional and Wellness Strategies for Neurological and Psychiatric Recovery From Post-COVID Syndrome and Post-acute Sequelae of COVID-19

**DOI:** 10.7759/cureus.51076

**Published:** 2023-12-25

**Authors:** Jeffrey Schaefer, Deepesh Khanna

**Affiliations:** 1 Foundational Sciences, Nova Southeastern University Dr. Kiran C. Patel College of Osteopathic Medicine, Clearwater, USA

**Keywords:** covid microbiome, kynurenine covid, omega-3 covid, vitamin c covid, vitamin d covid, post covid neurological, long-covid syndrome, pathophysiology of post-covid syndrome, neurological and psychiatric complications, post-covid-19 syndrome

## Abstract

The post-COVID syndrome was officially recognized as a disability under the Americans with Disabilities Act, indicating that this syndrome has made a significant impact on our populace. Also, post-acute sequelae of COVID-19 (PASC) is a term that describes the long-term health problems that some people experience after being infected with the virus that causes COVID-19. These problems can last for weeks, months, or even years, and can affect various parts of the body, such as the heart, lungs, brain, and blood vessels. This narrative review paper utilized the PubMed database to explore the pathophysiology of post-COVID syndrome's neurological and psychiatric symptoms and PASC and make therapeutic connections to the known mechanisms of various nutritional, supplemental, and wellness approaches. Searches were queried on the PubMed database between March 29 and April 16, 2022, using the phrases “long-covid,” “post-COVID syndrome,” “Vitamin D covid,” “vitamin C covid,” “omega-3 covid,” “kynurenine covid,” “whole-body hyperthermia,” “mushrooms immunity,” “n-acetyl cysteine covid,” “mushrooms cognition,” “sugar consumption inflammation,” and “covid microbiome.” Articles were screened for their relevance to the discussion of post-COVID syndrome's neurological and psychiatric pathophysiology at the discretion of the principal researcher. There were no limitations regarding publication years, but articles from 2005 to April 2022 were cited.

Micro-ischemic disease, neuropathy, autoimmune processes, mast-cell activation, and impaired blood-brain barriers have all been implicated in the pathological processes of this syndrome with varying degrees of supportive evidence. The common denominators, however, are inflammation and oxidative stress. Therefore, a beneficial approach to dealing with the complications of post-COVID syndrome would be to reduce the exacerbations of these common denominators with lifestyle and nutritional changes. Replenishing nutritional deficiencies, supplementing with N-acetylcysteine, decreasing consumption of refined sugars, preventing dysbiosis of the microbiome, performing exercises, increasing dietary intake of mushrooms, utilizing beneficial herbs such as rosemary, and increasing the core body temperature through whole-body hyperthermia seem to show potential for efficacy in this pursuit. Considering the safety and evidence-based connections of the therapies explored for dealing with the post-Covid syndrome, it could be of great benefit and of little harm to our patients to include these considerations in formulating post-Covid treatment plans.

## Introduction and background

According to the latest data from the WHO and the CDC, the COVID-19 pandemic has reached a staggering toll of confirmed cases and deaths worldwide as of the end of November 2023. The WHO reported that there have been almost 772 million confirmed cases of COVID-19, including 6.981 million deaths, globally. The CDC reported that in the United States of America, there have been 103 million confirmed cases of COVID-19 with 1.2 million deaths. These numbers are likely to increase as the pandemic continues to pose a serious threat to public health and safety [1,2].

Long COVID-19 and post-acute sequelae of COVID-19 (PASC) are terms used to describe the persistent and diverse health problems that some people experience after being infected with severe acute respiratory syndrome coronavirus 2 (SARS-CoV-2), the virus that causes COVID-19 [3]. These problems can last for weeks, months, or even years, affecting the heart, lungs, brain, blood vessels, etc [4-6]. Some common symptoms of PASC include fatigue, shortness of breath, chest pain, brain fog, anxiety, and depression. PASC can occur in anyone who has COVID-19, regardless of the severity of their initial infection or whether they were hospitalized or not. However, people who have severe COVID-19 or need intensive care may have a higher risk of developing PASC. The exact causes and mechanisms of PASC are still unknown, and there is no specific test or treatment for it. Therefore, it is important to prevent COVID-19 infection by getting vaccinated and following public health guidelines. People who have PASC should seek medical attention and may benefit from rehabilitation programs that can help them improve their physical and mental well-being.

Furthermore, Infection with COVID-19 often concludes with one or more persistent neurological symptoms that may last from a few weeks to several months [7]. Symptoms include fatigue, impaired memory, brain fog, headache, paresthesias, dysgeusia, and anosmia [8]. As of July 2021, post-COVID syndrome was officially recognized as a disability under the Americans with Disabilities Act (ADA). Despite this recognition, common treatment guidelines fail to address possible treatment models that include addressing the lifestyle and nutritional status of patients [9,10]. Compounding patterns of genetic disposition, sedentary living, adverse emotional states, ingestion of pro-inflammatory foods and chemicals, and deficiencies of key nutrients present as a driver of decreased health span and increased susceptibility to a vast majority of illnesses, including post-COVID syndrome [11-13]. Promoting general health through supporting the required intakes of the body should not fail to be a model of standard care for patients [14,15]. The purpose of this narrative review is to make evidence-based connections between the proposed mechanisms of persistent post-COVID neurological symptoms and various nutritional and lifestyle interventions.

## Review

Methodology

**Figure 1:**
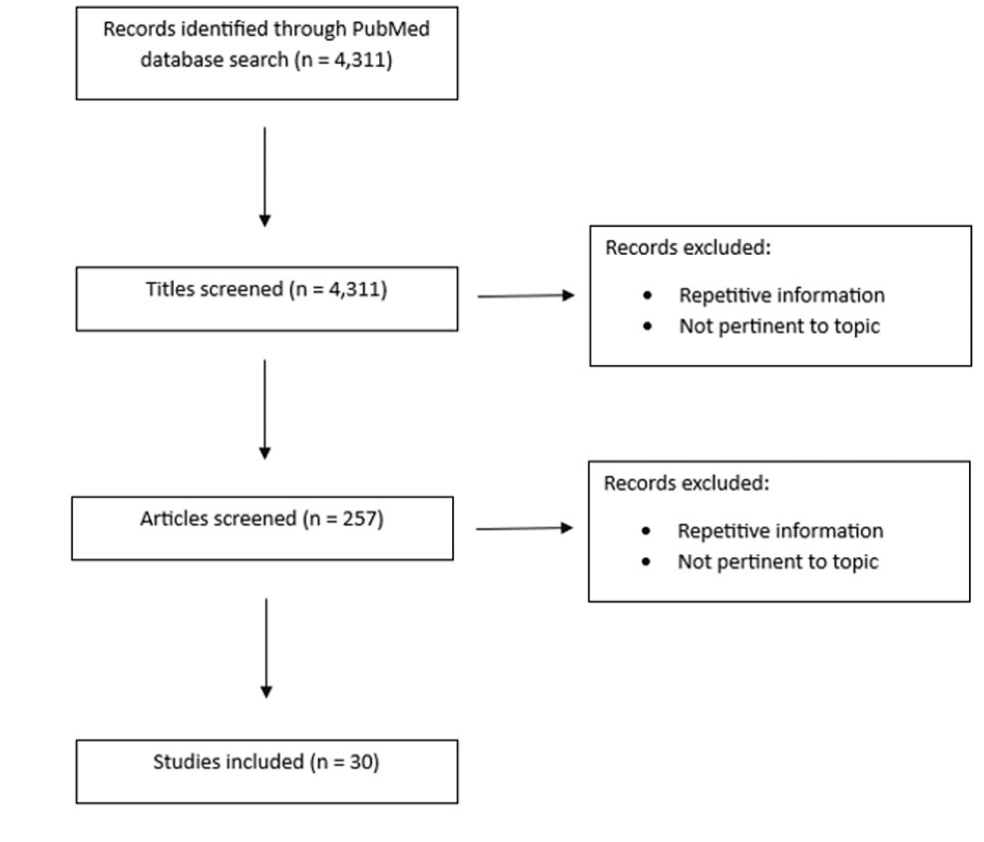
PRISMA diagram

Searches were made on the National Institute of Health PubMed database between March 29 and April 16, 2022, using the phrases “long-covid,” “post-COVID syndrome,” “Vitamin D covid,” “vitamin C covid,” “omega-3 covid,” “kynurenine covid,” “whole-body hyperthermia,” “mushrooms immunity,” “n-acetyl cysteine covid,” “mushrooms cognition,” “sugar consumption inflammation,” and “covid microbiome.” Articles were screened for their relevance to the discussion of post-COVID syndrome's neurological and psychiatric pathophysiology at the discretion of the principal researcher. There were no limitations regarding publication years, but articles from 2005 to 2023 were cited. Search data is summarized in Figure 1.

Discussion

Demographics and Mechanisms of Long-Haul COVID-19's Neurological Symptoms

**Figure 2:**
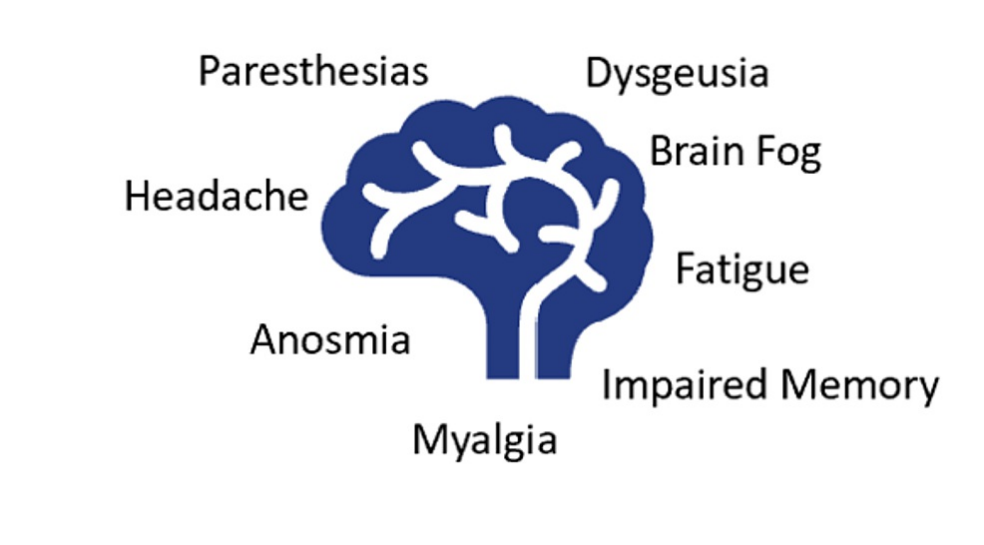
Neurological manifestations of post-COVID syndrome

Post-COVID syndrome has been characterized as the continuation of symptoms for greater than three weeks after diagnosis. The prevalence of post-COVID syndrome in the general population has been estimated to be about 10-35% while reaching up to 85% in the hospitalized [7]. The mean age of patients has been measured to be about 43.2 ± 11.3 years [8]. An increase in incidence has been reported in the female population with a female-to-male ratio of 2.3:1, which closely resembles the gender distribution of autoimmune disease [8]. It has also been observed that in the populations experiencing post-COVID syndrome, there was a higher prevalence of both autoimmune disease and depression/anxiety compared to the normal population [8]. Symptoms of post-COVID syndrome with their prevalence include fatigue (85%), brain fog (81%), headache (68%), numbness/tingling (60%), dysgeusia (59%), anosmia (55%), and myalgia (55%) [8]. Deficits have also been found in both short-term memory and attention [8]. An overview of these symptoms is referenced in Figure 2.

The mechanisms behind the pathophysiology of post-COVID syndrome are not fully clear, but a few theories have sprung up. Transcranial Doppler ultrasound has shown intracerebral microemboli in the brains of those experiencing the virus, which possibly suggests ischemia-related encephalopathy [16]. Pathological reports have also shown large amounts of megakaryocytes or platelet progenitor cells in the cortical capillaries of patients showing further support for the idea that endothelial dysfunction and neuro-ischemia have occurred from the virus [17]. Post-COVID myalgias have been theorized to be a result of chronic oxidative stress, low-grade inflammation, and impaired heat shock proteins [18].

Dysgeusia and anosmia are common complaints of COVID-19 as well as post-COVID syndrome. Neurotropism for the olfactory and trigeminal nerves by the virus via spike protein-ACE2 interactions and direct cytotoxicity of the virus to the taste buds have been implicated in this pathogenesis. Zinc, which is an important mineral for the immune system, also has roles in taste, and hypozincemia could potentially be a cause of extended periods of dysgeusia [19]. Chronic inflammation as a result of IL-6 may also have a role in preventing the renewal of taste buds and olfactory nerves [19].

Autoimmune mechanisms have been linked to theories on the effects of the spike protein. Some studies have found a potential for antigenic epitopes of the protein to be shared with those of common chaperone proteins resulting in autoimmunity of the endothelial cells [20]. Spike protein-ACE2 interactions on neural glial cells could possibly lead to downstream mast cell activation and inflammation [20]. Mast cells are perivascularly organized in the brain and have high concentrations in the hypothalamus. A compelling part of this mast-cell activation theory is that the has characteristically similar symptomology to post-COVID syndrome. The spike protein also has actions on toll-like receptors (TLRs) such as TLR2, which induces pro-inflammatory cytokine upregulation, and TLR4, which increases the expression of ACE2. Activation of these TLRs can potentially lead to the activation of mTOR in the brain, which has been associated with various neuropsychiatric diseases [20].

Researchers have found that 70% of SARS-CoV-2 proteins affect endothelial integrity [21]. Of these proteins, the most significant are non-structural, such as nsp2, nsp5_c145a, and nsp7, which have been found to induce the expression of hemostatic von-Willebrand factor and inflammatory cytokines. In-vitro models of human umbilical vein endothelial cells exposed to these non-structural proteins showed a decrease in endothelial function [21].

Overall, degradation of the blood-brain barrier via viral non-structural proteins and neurotrophic translocation via spike proteins have appeared to be the main ways in which the virus invades the central nervous system (CNS). From here the continuing basis of damage appears to be inflammatory with suggestions that up-regulated mTOR, aberrantly activated mast cells, autoimmune reactions, and increased permeability of the blood-brain barrier are implicated in the pathogenesis. Considering the diversity of genotypes and phenotypes of both the virus and humans, careful consideration of the underlying cause of a patient’s post-COVID syndrome needs to be done. Suspected deficits in the blood-brain barrier can be tracked with both biochemical and radiographic methods such as assaying the cerebrospinal fluid (CSF) for CSF/plasma albumin ratios and levels of soluble platelet-derived growth factor receptor β or using dynamic contrast-enhanced MRI [22]. Neural autoimmune reactions could also warrant examination of the CSF for the presence of self-reactive immunoglobulins and inflammatory biomarkers like IL-6, which have already been noted in patients with an active viral infection [20]. Observational studies need to be conducted in patients suffering from post-COVID syndrome studying the collective integrity of the cohort’s blood-brain barriers and CSF constitution for signs of inflammatory and autoimmune processes. A review of the proposed etiologies is presented in Table 1. 

**Table 1:** Proposed etiologies of post-COVID syndrome

Proposed etiologies	Supportive data
Autoimmune processes	Female to male incidence of 2.3:1 mirrors the incidence of autoimmune disease [8]. Higher prevalence of existing autoimmune disease in post-covid syndrome populations than in control populations [8]. Spike protein antigenic epitopes bear resemblance to endothelial chaperone proteins [20].
Micro-ischemic disease	Intracerebral microemboli detected in patients with severe virus with intracranial ultrasound [16]. Megakaryocytes detected in cortical capillaries [17].
Impaired blood-brain barrier	Viral non-structural proteins inducing the expression of Von-Willebrand factor and cytokines [21]. Impaired function in human umbilical endothelial cells exposed to viral non-structural proteins [21]. Megakaryocytes detected in cortical capillaries [17].
Neuropathy	Neurotropism of virus to trigeminal and olfactory ganglia via Spike-ACE2 Interactions [19]. Spike-TLR2 Interactions upregulating TLR4, ACE2, mTOR, and cytokine production [20].
Mast cell activation syndrome	Similar Symptomology to post-COVID-19 Syndrome [20]. Potential for Spike-ACE2 interactions at glial cells to activate mast cells [20].

Functional Approaches to Dealing with Post-COVID Syndrome

Considering the known pro-inflammatory and pro-oxidative stress mechanisms of COVID-19, care should be taken so that the patients’ lifestyles do not exacerbate these disease processes. Chronic oxidative stress typically wears out the body’s stores of antioxidants and antioxidative cofactors. Therefore, patients should be advised to ensure that they are getting sufficient levels of essential antioxidative vitamins such as Vitamin A, Vitamin C, and Vitamin E [23,24]. Vitamin C has been used throughout the pandemic as an adjunctive therapy. A small hospital-based study has demonstrated that patients on fractionated oxygen receiving an IV drip of 1 gram of the vitamin every eight hours for three days had a 9% decrease in mortality and a 5% decrease in the need for ventilation [24]. Supporting the body's endogenous antioxidant glutathione is also important for combating chronic oxidative stress. Alcohol and tobacco consumption are important sources of glutathione depletion, and levels of consumption should be potentially considered in patients. N-acetylcysteine is a modified version of cysteine with an increased capacity for replenishing glutathione levels. N-acetylcysteine has the capacity to decrease symptoms of depression and other psychiatric illnesses such as obsessive-compulsive disorder and anxiety by promoting a healthier glutamate balance in the brain [25,26]. Selenium and riboflavin are both important factors for utilizing and reducing glutathione, respectively. Data has already come out to show that selenium deficiency in COVID-19 patients was associated with worse disease outcomes [27]. Selenium is also important for the formation of selenoproteins, which tend to be antioxidative and immunomodulatory in nature.

Decreased levels of vitamin D have also been strongly linked to worse outcomes and increased susceptibility to COVID-19 [28]. Evidence has amounted in various ways: countries closer to the equator have lower mortality rates, increased cytokine storms and need of care for patients with deficiency, increased rates of ventilation in the deficient, decreased inflammatory biomarkers in those receiving supplementation, and decreased lymphocyte counts and hemoglobin in the deficient [28]. Vitamin D supplementation may also have a role in combating the potential autoimmune mechanisms of long-COVID. Studies have found that the supplementation of 2000 IU/day for a period of five years reduced the risk of autoimmune diseases by 22% [23]. Mechanisms of this action could be linked to its ability to inhibit IL-2 and suppress IL-12, IL-6, IFN-ɣ, and TNF [23].

Another aspect of curbing inflammatory and autoimmune processes is omega-3 fatty acid supplementation. Studies have shown that supplementation of 460 mg of eicosapentaenoic and 380 mg of docosahexaenoic acid daily over a period of five years resulted in a 15% decrease in autoimmune disease risk. ­Omega-3 fatty acids have roles in generating anti-inflammatory eicosanoids, preventing the formation of pro-inflammatory eicosanoids, and inhibiting CRP, TNF-ɑ, IL-1b, and IL-6 [23]. Omega-3 supplementation was also found to decrease markers of respiratory acidosis in hospital-bound COVID-19 patients, improve endothelial and microcirculatory function, increase tissue hemoperfusion, and increase capillary flow [29]. Omega-3 fatty acids also have essential roles in neurological health. Studies have shown that supplementation can mitigate damage from traumatic brain injury by protecting the myelin sheaths and thus signal transduction of nerves in the white and grey matter of the brain [30]. Mechanisms are attributed to the protection of myelin basic protein and the protection of oligodendrocytes via suppression of microglial activation [30].

The suppression of microglia may also be a significant property for the amelioration of post-COVID neurological symptoms if the mast-cell activation syndrome theory is correct. Improvement of endothelial and microcirculatory function may also prove to be beneficial for patients whose blood-brain barrier had been compromised following infection. Zinc supplementation as well as suppression of IL-6 may also be significant in combating post-COVID dysgeusia and replenishing taste buds and olfactory nerves as mentioned earlier in the paper. Cobalamin or vitamin B12 is also essential for maintaining the health of myelin sheaths and deficiencies should be considered in patients with dietary risk factors when dealing with patients experiencing long-COVID syndrome.

Excessive sugar consumption may also contribute to increased inflammatory states. Studies have shown in insulin-sensitive individuals that C-reactive protein levels increase progressively with blood glucose [31]. High glucose and fructose diets result in microbiome changes leading to increased gut permeability, inflammation, and adiposity [32]. Increasing levels of body fat lead to and sustain obesity, which has been a significant comorbidity of COVID-19 [33].

Post-COVID patients were in total found to have lower levels of butyrate-producing bacteria such as *Bifidobacterium pseudocatenulatum* and *Faecalibacterium pastnitzii* [28]. It was also found that patients with post-COVID neuropsychiatric symptoms and fatigue were likely to have higher amounts of nosocomial bacteria such as *Clostridium innocuum* and *Actinomyces naeslundii *[34]. The diversity of the gut flora upon illness was shown to be a predictive factor for the occurrence of long-term COVID-19 symptoms. Treatment of patients with oral probiotics and counseling them on dietary factors that shape the microbiome composition could be beneficial to their outcomes.

Kynurenine is a by-product of tryptophan metabolism and has a depressogenic effect in the brain where it converts to quinolinic acid and contributes to oxidative damage [35]. Higher levels of kynurenine have been associated with increased mortality from infection with COVID-19 [36]. Skeletal muscle expresses an enzyme, PGC-1ɑ1, which converts kynurenine into kynurenic acid, decreasing its ability to diffuse into the brain and increasing resilience to depression [35]. Exercise directly increases levels and activity of PGC-1ɑ1 in skeletal muscles.

Mushrooms are a food group with a wide variety of active compounds with immunomodulatory, anti-inflammatory, anti-oxidative, and nootropic properties [37,38]. An epidemiological study has found that older adults eating more than two servings of mushrooms per week had a decreased risk of mild cognitive decline independent of possible confounding variables [37]. *Hericium erinaceus* full spectrum extract was found to be an effective neurogenesis-inducing anti-depressant in animal models during a four-week treatment period [38]. Mechanisms include elevated neurogenesis-related proteins such as brain-derived neurotrophic factor, synaptophysin, etc. as well as reduced neuroinflammation [39]. Mushrooms are also potent sources of antioxidants such as ergothioneine and glutathione, which protect against the deleterious effects of oxidative damage [40]. Increased general consumption of mushrooms and specific consumption of *Hericium erinaceus* fruiting body or its full-spectrum extracts could potentially be helpful in ameliorating the effects of post-COVID cognitive decline, depression, oxidative stress, and neuropathy.

Rosemary or *Salvia rosmarinus* is a commonly appreciated herb and tea. Studies have indicated that its diterpene, carnosic acid has the potential to decrease neuro-inflammation by its lipophilic property that allows it to cross the blood-brain barrier and its ability to downregulate the NLRP3 inflammasome via NRF2 activation [41].

Sauna bathing is an increasingly popular modality of health and wellness. Inducing whole-body hyperthermia of 38-39 ℃ was found to be beneficial for patients with depression [42]. Some research has shown that this benefit may be linked to the activation of integumentary temperature-sensitive ion channels inducing signaling pathways that ultimately synapse in serotonergic neurons in the brainstem [42]. Whole-body hyperthermia has also been found to drastically increase the expression of heat shock proteins, which effectively increase the resilience of the body toward inflammation [43]. Heat shock protein dysregulation, as mentioned earlier, has been theorized to be a possible source of post-COVID myalgias and can potentially play a role in benefiting inflammatory-induced depression. Increasing the expression of heat shock proteins through whole-body hyperthermia is also a potentially viable way to increase exercise tolerance for people habituated to sedentary lifestyles [43]. Care should be taken with recommendations for whole-body hyperthermia via sauna bathing or hot tubbing as it may be contraindicated for people with certain conditions.

All of these approaches have been tabulated in Table 2.

**Table 2:** Functional approaches and supportive evidence

Explored approach	Supportive data
Vitamin A, C, and E supplementation	Ensure sufficiency of essential antioxidant vitamins. Patients on fractionated oxygen receiving an IV drip of 1 gram every eight hours for three days had a 9% decrease in mortality and a 5% decrease in the need for ventilation [23].
N-acetylcysteine	Support endogenous antioxidant system via restoration of glutathione. Promotes a healthier glutamate balance via action on the cysteine-glutamate exchanger [24]. Proven benefits for multiple psychiatric illnesses [31].
Selenium	Important cofactor for glutathione peroxidase. Necessary to form beneficial selenoproteins. Deficiency is associated with worse disease outcomes [26].
Vitamin D deficiency	Associated with worse disease outcomes and increased susceptibility [21]. Countries closer to the equator have lower mortality rates [27]. Increased cytokine storms and need for care for patients [21]. Increased rates of ventilation in the deficient [27]. Decreased inflammatory biomarkers in those receiving supplementation [27]. Decreased lymphocyte counts and hemoglobin in the deficient [27]. Supplementation of 2000 IU/day for five years reduced the risk of autoimmune diseases by 22% [28]. Ability to inhibit IL-2 and suppress IL-12, IL-6, IFN-ɣ, and TNF [28].
Omega-3 fatty acids	460 mg of eicosapentaenoic and 380 mg of docosahexaenoic acid daily over five years resulted in a 15% decrease in autoimmune disease risk [28]. ­Generate anti-inflammatory eicosanoids, preventing the formation of pro-inflammatory eicosanoids, and inhibiting CRP, TNF-ɑ, IL-1b, and IL-6 [28]. Decrease markers of respiratory acidosis in hospital-bound COVID-19 patients, improve endothelial and microcirculatory function, increase tissue hemoperfusion, and increase capillary flow [29]. Can mitigate damage from traumatic brain injury by protecting the myelin sheaths and signal transduction in the white and grey matter [37]. Protection of myelin basic protein and oligodendrocytes via suppression of microglial activation [30].
Zinc	Deficiency associated with post-COVID dysgeusia [19]. Important roles in genetic replication, gene transcription, innate immunity, and adaptive immunity.
Vitamin B12	Maintains the health of myelin sheaths.
Excessive sugar	Insulin-sensitive individuals have C-reactive protein levels that increase progressively with blood glucose [31]. High glucose and fructose diets result in microbiome changes leading to increased gut permeability, inflammation, and adiposity [32].
Reversing obesity	Significant comorbidity of COVID-19 [33]. Inflammatory and compressive nature of increased adiposity on the body.
The microbiome of affected patients	Lower levels of butyrate-producing bacteria such as *Bifidobacterium pseudocatenulatum* and *Faecalibacterium pastnitzii* [28]. Increased likelihood of post-COVID-19 neuropsychiatric symptoms and fatigue correlated to higher levels of *Clostridium innocuum* and *Actinomyces naeslundii* [34]. The diversity of the gut flora upon illness shown to be a predictive factor for the occurrence of long-COVID symptoms [34].
Mushrooms	Wide variety of active compounds with immunomodulatory, anti-inflammatory, anti-oxidative, and nootropic properties [37,38]. Older adults eating more than two servings of mushrooms per week had a decreased risk of mild cognitive decline [38]. *Hericium erinaceous* full spectrum extract was shown to be an effective neurogenesis-inducing anti-depressant in animal models. Elevates brain-derived neurotrophic factor, synaptophysin, and decreases neuroinflammation [39]. Potent sources of antioxidants such as ergothioneine and glutathione [40].
Exercise and kyneurenine	Exercise induces PGC-1ɑ1 in skeletal muscle converting kynurenine into kynurenic acid, thus decreasing its ability to diffuse into the brain and increasing resilience to depression [35]. Kynurenine is a by-product of tryptophan metabolism and has a depressogenic effect in the brain where it converts to quinolinic acid and contributes to oxidative damage [35]. Higher levels of kynurenine have been associated with increased mortality from infection with COVID-19 [36].
Rosemary	Diterpene constituent, carnosic acid, crosses the blood-brain barrier and downregulates the NLRP3 inflammasome via NRF2 activation [41].
Sauna and whole-body hyperthermia	Inducing whole-body hyperthermia of 38-39 ℃ was found to be beneficial for patients with depression [42]. A large increase in heat shock protein expression, a potentially viable way to increase exercise tolerance for people habituated to sedentary lifestyles [43].

## Conclusions

The exact mechanisms behind long-COVID neurological symptoms remain elusive. Micro-ischemic disease, neuropathy, autoimmune processes, mast-cell activation, and impaired blood-brain barriers have all been implicated in the pathological processes of the syndrome. The common denominators, however, are inflammation and oxidative stress. Therefore, the functional approach to dealing with long-COVID syndrome would be to reduce these common denominators with lifestyle and nutritional changes. Despite a lack of evidence for high-quality standardized control trials for many of the functional therapies explored in this paper to explicitly treat post-COVID symptomology, replenishing nutritional deficiencies, supplementing with N-acetylcysteine, decreasing the consumption of refined sugars, harmonizing the microbiome, exercising, consuming mushrooms, consuming rosemary, and utilizing modalities to reach whole-body hyperthermia are all generally very safe interventions that are already a part of individual wellness routines worldwide. Considering the safety and evidence-based connections of the therapies explored for dealing with the post-COVID syndrome, it could be of great benefit and of little cost to our patients to take a holistic approach to formulating post-COVID treatment plans. Difficult circumstances are often present as catalysts for making great change. Physicians, utilizing this truth by providing counseling and motivation to our patients about healthy living, can present an excellent opportunity to ease ailments and increase the well-being of the patients.
